# Comparison of Distortion-Product Otoacoustic Emissions Measured in the Same Subjects Using Four Commercial Systems

**DOI:** 10.3390/jcm14228184

**Published:** 2025-11-18

**Authors:** Edyta Pilka, Henryk Skarżyński, W. Wiktor Jedrzejczak

**Affiliations:** 1 Institute of Physiology and Pathology of Hearing, Mochnackiego 10 Street, 02-042 Warsaw, Poland; 2 World Hearing Center, Mokra 17 Street, 05-830 Kajetany, Poland

**Keywords:** DPOAE, measurement repeatability, test–retest

## Abstract

**Background/Objectives**: Distortion-product otoacoustic emissions (DPOAEs) are suited to longitudinal cochlear assessment, but inter-system differences may confound interpretation across clinics. This study compared DPOAE outcomes across four commercial systems and evaluated their within-session repeatability. **Methods**: Adults with normal hearing (84 ears) were tested using the HearID DP (Mimosa Acoustics), SmartDPOAE (Intelligent Hearing Systems), Eclipse DPOAE20 (Interacoustics), and Echoport ILO 292 USB I (Otodynamics). DPOAEs were recorded at 1, 1.5, 2, 3, 4, 6 and 8 kHz using a criterion of ≥6 dB signal-to-noise ratio. Two measurements per ear were obtained, with the probe repositioned between sessions. **Results**: All systems showed similar frequency response profiles but substantially different absolute values. Between-system amplitude differences were smallest at 1.5–4 kHz and largest at 6 kHz. Noise floors varied considerably: HearID DP and SmartDPOAE were best (lowest) while Echoport ILO 292 USB I and Eclipse DPOAE20 were worst (highest), with inter-system differences most prominent between 1.5 and 4 kHz. HearID DP achieved the highest detection rates (84/84 ears at key frequencies). Test–retest reliability was good across all systems. The standard error of measurement varied from 0.99 to 2.88 dB, the smallest being the HearID DP. Within-session differences were typically ≤2 dB, with the best repeatability between 1.5 and 6 kHz. **Conclusions**: Despite similar frequency responses, clinically significant differences exist between DPOAE systems in terms of noise floors, signal-to-noise ratios, and response amplitudes. Inter-device variations frequently exceeded minimum detectable change values, meaning that DPOAE devices cannot be considered clinically interchangeable. These findings underscore the need for industry-wide standards to enable reliable cross-clinic comparisons.

## 1. Introduction

Otoacoustic emissions (OAEs) are weak signals generated in the cochlea of the inner ear and can be recorded using a probe equipped with a sensitive microphone placed in the external ear canal. Since even minor hearing disturbances can affect OAEs and cause drops in the emitted signal, the technique is a powerful diagnostic tool for objectively assessing the condition of the cochlea, e.g., [1,2,3,4,5].

One common type of emission is the distortion product otoacoustic emission (DPOAE) [6,7,8,9], which is widely used clinically to evaluate hair cell function (e.g., in cases of Meniere disease and sudden sensorineural hearing loss) [10]. DPOAEs are particularly valuable for longitudinal monitoring of cochlear status, with serial measurements used clinically to detect ototoxic damage during treatment with cisplatin, aminoglycosides, and other ototoxic medications [11,12,13]. Early detection of ototoxicity through DPOAE monitoring enables intervention before changes become apparent on conventional audiometry, potentially preventing further hearing deterioration [14]. However, DPOAE measurements can be affected by factors such as stimulation parameters, type of probe, or signal analysis algorithm [15,16].

For ototoxicity surveillance and other longitudinal monitoring applications to be effective, high test–retest reliability and inter-instrument comparability are critical requirements, especially if a patient undergoes repeated testing over time, possibly with different equipment at different clinical sites [17]. Measurement variability can then mask true ototoxic changes or generate false positives, compromising clinical decision-making. As a benchmark, one meta-analysis has established that a DPOAE level shift of more than around 5 dB at one or more frequencies points to clinically significant cochlear damage [17]. Beyond instrumentation differences, probe insertion technique represents another significant source of measurement variability in serial DPOAE monitoring. Variations in probe placement—depth, angle, and fit—can alter ear canal acoustics through changes in standing wave patterns and resonances [18]. In addition, inconsistent probe insertion affects the acoustic seal and attenuation of environmental noise, which in turn impacts signal-to-noise ratio and measurement reliability [19]. The interaction between probe position and ear canal geometry can produce frequency-specific variations in DPOAE amplitude of several decibels, potentially obscuring or mimicking true cochlear changes [20]. These variabilities explain why many papers reporting DPOAE measurements using a given piece of equipment focus on achieving good reproducibility, which is the core of all tests of reliability, e.g., [20,21,22,23,24,25,26]. Whereas reproducibility is scientifically desirable, the complication is that different diagnostic centers often use different measuring equipment. Unfortunately, there are few works comparing such equipment, even though the literature has shown that differences between measuring equipment can be major, and can be sufficiently large to prevent subtle changes occurring in the cochlea from being detected [27,28,29,30].

The rationale for the current study is to help fill the gap concerning differences between OAE systems in terms of their measurement repeatability. Accurate OAE testing underpins modern newborn hearing screening programs and clinical practice, yet often it proceeds with the hidden assumption that different OAE devices provide interchangeable results. That assumption is risky: measurement error can mask early cochlear damage or even generate false referrals [31]. Although the ability to distinguish true biological changes from measurement variability is fundamental to effective ototoxicity detection and other monitoring applications [12], there is limited data from which audiologists and researchers can confidently select equipment, set pass/fail criteria, and accurately interpret longitudinal changes.

The main purpose of this study was to compare DPOAEs measured from 1 to 8 kHz using four commercial systems: HearID DP (version 6.1.7601, Mimosa Acoustics, Champaign, IL, USA), SmartDPOAE (version 4.70, Intelligent Hearing Systems, Glenvar Heights, FL, USA), Eclipse DPOAE20 (version 1.03, Interacoustics, Middelfart, Denmark), and Echoport ILO 292 USB I (version 6.41.27.33, Otodynamics, Hatfield, UK). An additional objective was to evaluate the repeatability of measurements when the probe was re-fitted.

## 2. Materials and Methods

### 2.1. Participants

The measurements were performed on 42 adults (84 ears) aged 30.4 ± 9.4 years (35 females of 31.7 ± 10.5 years and 7 males of 27 ± 4 years). All subjects were in good general health and reported no history of otological disease or exposure to ototoxic drugs or loud noise.

### 2.2. Procedures

Inclusion criteria were: normal otoscopic findings; normal middle ear status (type A tympanograms and well-defined compliance maximum of not less than ±100 daPa; static compliance of 0.3–1.3 mmho; and normal ipsilateral and contralateral acoustic stapedial reflexes at 0.5, 1.0, 2.0, and 4.0 kHz [32,33]); and normal hearing sensitivity (pure-tone air conduction thresholds better than or equal to 20 dB HL at 0.125, 0.25, 0.5, 1, 1.5, 2, 3, 4, 6, and 8 kHz and no air-bone gap between 0.125 and 8 kHz [34]).

Each subject was tested in a sound-treated booth and avoided strong noise prior to testing. Middle ear function was determined by tympanometry at 226 Hz using the Madsen Zodiac 901 tympanometer (GN Otometrics, Taastrup, Denmark). Pure tone audiometry was assessed using a Madsen Astera (GN Otometrics, Taastrup, Denmark) connected to Sennheiser HDA-200 headphones (Wannebostel, Germany). Bone conduction thresholds were measured using a RadioEar B-71 bone vibrator (Pittsburgh, PA, USA). Hearing thresholds were determined using the modified Hughson–Westlake method [35]. In this procedure, the test tone is first administered at a level the patient can clearly hear; the intensity of the tone is then reduced by fixed increments until the patient stops responding. The intensity is then increased until the patient again responds. The steps are repeated until at least two response levels are identical.

DPOAEs were measured using four systems: HearID DP (version 6.1.7601, Mimosa Acoustics, Champaign, IL, USA), SmartDPOAE (version 4.70, Intelligent Hearing Systems, Glenvar Heights, FL, USA); Eclipse DPOAE20 (version 1.03, Interacoustics, Middelfart, Denmark); and Echoport ILO 292 USB I (version 6.41.27.33, Otodynamics, Hatfield, UK). Table 1 shows the measurement systems, the versions used in the study, and the DPOAE stimuli parameters.

DPOAEs were evoked by two tones (denoted by F1 and F2), and responses were measured at the nonlinear distortion product frequency 2F1 − F2. Other measurement settings used were the same as the system defaults. However, it was not possible to set the exact same frequencies for the four systems, so rounded values as listed in Table 1 were used for comparisons. Each probe was used with ear tips native to the system; this meant the HearID DP system carried a probe with foam ear tips, while the other systems were equipped with silicone ear tips. All systems used in situ SPL calibration.

Each recording session consisted of the two measurements for each subject’s ear in each system. The second measurement for each ear was made after totally removing and refitting the probe. The order of systems and choice of ear to test were random. To assess the presence of DPOAE signals, the criterion of an SNR greater than 6 dB at 3 or more of the 7 tested frequencies was used [36]. In this context, it is worth noting that there are other alternative criteria for assessing the presence of DPOAE signals [37,38,39,40]. In our work, DPOAE recordings required a minimum of two complete sweeps across all test frequencies, with automatic termination occurring once at least three frequencies exhibited SNRs ≥ 6 dB.

The data used, collected, and analyzed in this study are available as a Supplementary Materials.

Each subject gave written informed consent prior to participation. The research procedures were approved by the Ethics Committee of the Institute of Physiology and Pathology of Hearing, Poland (approval no. IFPS:KB/13/2014, date 13 November 2014).

All analyses were made in Matlab (version 2024b, MathWorks, Natick, MA, USA) and in Statistica (version 7.1, StatSoft. Inc., Tulsa, OK, USA). For all measured parameters, the statistical significance of mean differences was evaluated using repeated-measures analysis of variance (rmANOVA). Post hoc tests were conducted using a *t*-test when the data fulfilled a criterion of normality, otherwise a Wilcoxon rank-sum test was used. A Shapiro–Wilk test was used to check the normality of variables. Benjamini–Hochberg corrections were applied to allow for multiple comparisons.

The reliability of measurements was evaluated by the two-way random-effects intraclass correlation coefficient (ICC) and the standard error of measurement (SEM). SEM was calculated as $STD\times \sqrt{1-ICC}$, where STD is the standard deviation of the test and retest DPOAE estimate. Larger SEM values indicate poorer reliability. Based on the SEM, the minimum detectable change (MDC) can be determined: if the confidence interval is set at 95%, then the MDC is given as $\pm 1.96\times \sqrt{2}\times SEM$.

## 3. Results

**Figure 1:**
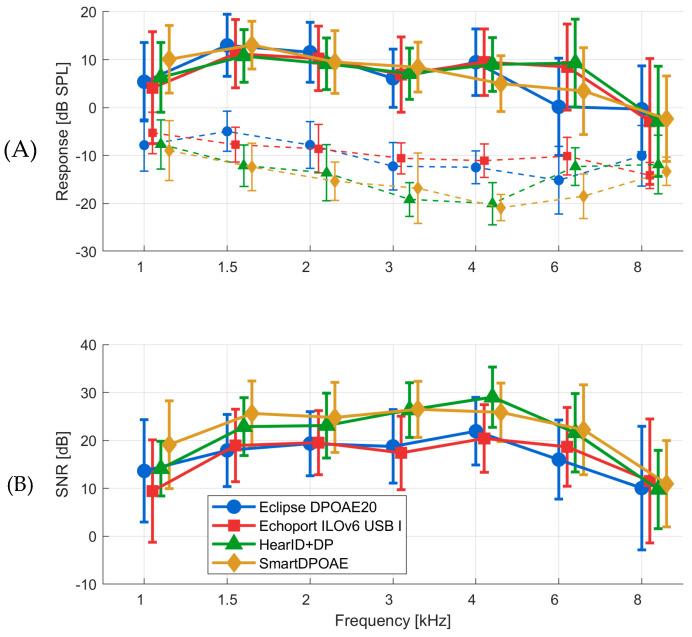
(**A**): DPOAEs measured by each of the four systems. Response levels—solid lines; noise floor—dashed lines. (**B**): Signal to noise ratios (SNRs); whiskers indicate standard deviations.

All analyses of DPOAE measurements were presented for both ears combined (*N* = 84 ears from 42 participants). Figure 1A shows the DPOAE response levels and noise floors across the 1–8 kHz frequency range for all four systems. All systems demonstrated similar frequency response profiles, with DPOAE amplitudes generally increasing from 1 kHz and reaching peak responses in the 2–4 kHz region. SmartDPOAE and Eclipse DPOAE20 showed lower response amplitudes, particularly at higher frequencies.

Substantial differences were observed in noise floors between systems. The HearID DP and SmartDPOAE systems demonstrated the lowest noise floors, while the Echoport ILO 292 USB I showed the highest noise floor across most frequencies, with differences exceeding 10 dB over 1.5–4 kHz.

The signal-to-noise ratios (SNRs) are presented in Figure 1B. Because of their lower intrinsic noise levels, the HearID DP and SmartDPOAE systems achieved higher SNRs than the other two; differences between these two systems and the others reached about 6 dB between 1.5 and 4 kHz. Despite the SNR differences, most individual measurements resulted in about the same pass/fail outcomes when applying the 6 dB SNR criterion.

**Figure 2:**
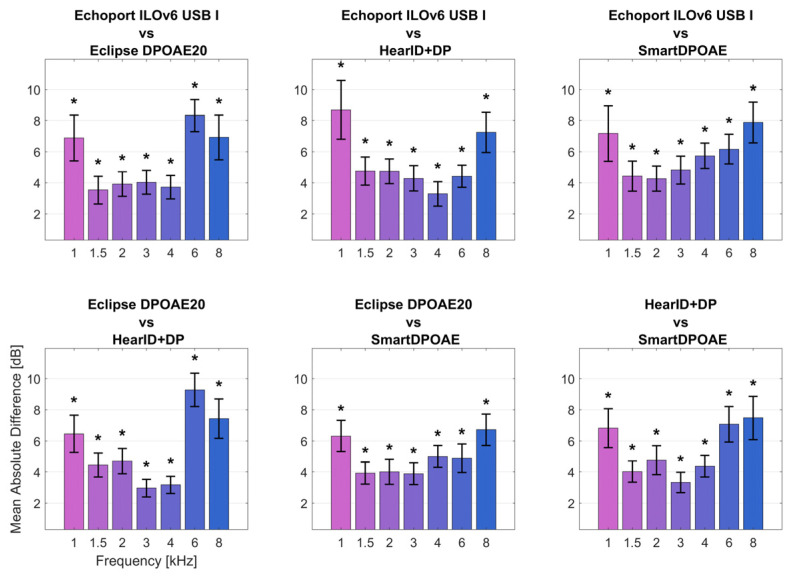
Absolute average differences in DPOAE response levels between the four measurement systems. Error bars show 95% confidence intervals. Asterisks indicate significant differences (at *p* < 0.05) after Benjamini–Hochberg correction for multiple comparisons. The different colors of the bars represent different frequencies.

Figure 2 illustrates the absolute average differences in DPOAE response levels between all possible system pairs. The smallest between-system differences occurred in the 1.5–3 kHz range, where mean differences were typically less than 4 dB. The largest differences were observed at 6 kHz, where some system pairs showed mean differences exceeding 9 dB. Significant differences (*p* < 0.05, with correction for multiple comparisons) were found at multiple frequencies, being highest at 1, 6, and 8 kHz, as indicated by the asterisks in Figure 2. The frequency range from 1.5 to 3 kHz showed the best inter-system agreement, while the 6–8 kHz region had the poorest agreement between devices.

Table 2 presents the reliability metrics for repeated measurements after probe repositioning, including intraclass correlation coefficients (ICC), standard error of measurement (SEM), and minimum detectable change (MDC) values. The number of ears meeting the 6 dB SNR criterion (*N* pass) varied by system and frequency, as shown.

ICC values generally ranged from 0.86 to 0.97 across all systems and frequencies. The HearID DP system showed the highest ICC values in the 1–1.5 kHz and 3–6 kHz range (0.94–0.97), with strong reliability at 4 kHz (ICC = 0.97). The SmartDPOAE system performed best at 6 kHz (ICC = 0.96), while the Eclipse DPOAE20 showed its highest reliability at 4 kHz (ICC = 0.93) and Echoport ILOv6 USB at 3 kHz (ICC = 0.92).

SEM values varied considerably across systems and frequencies. The HearID DP system demonstrated the lowest SEM values in the 1–1.5 kHz and 3–4 kHz range, with the best performance at 4 kHz (SEM = 0.99 dB). The SmartDPOAE system showed its lowest SEM at 3 kHz (1.32 dB) and 4 kHz (1.34 dB). The Eclipse DPOAE20 system showed its lowest SEM at 4 kHz (1.70 dB) and Echoport ILOv6 USB I at 4 kHz (1.42 dB). Overall SEM values ranged from approximately 0.99 dB to 2.88 dB across all systems.

MDC values, representing the 95% confidence interval for detecting real changes, ranged from 2.74 to 7.98 dB. The HearID DP system achieved the smallest MDC values at 4 kHz (2.74 dB), indicating better sensitivity for detecting small changes at these frequencies.

The HearID DP system consistently achieved the highest number of ears meeting the 6 dB SNR criterion across most frequencies, with detection rates of 84/84 ears at 1.5, 3, and 4 kHz. The SmartDPOAE and Eclipse DPOAE20 systems showed similar detection patterns, while the Echoport ILO 292 USB I generally had lower detection rates, particularly at the frequency extremes.

Mean within-session differences in DPOAE amplitude after probe repositioning were generally ≤2 dB across most frequency–system combinations, demonstrating good overall repeatability. The poorest repeatability typically occurred at 1 kHz and 8 kHz, with some systems showing differences up to 3.8 dB at these frequencies. The frequency range of 1.5–6 kHz consistently showed the best repeatability across all systems tested.

## 4. Discussion

In this study we assessed DPOAEs in normal-hearing adults using four commercial recording systems covering the 1–8 kHz band and taking two runs per ear, with the second run done after re-fitting the probe. Although all devices produced similar frequency responses and showed good between-run repeatability, they differed in background-noise levels and frequency responses (especially at higher frequencies). Although the outputs were repeatable within each device, they were not the same between devices.

Comparisons of all the systems showed that DPOAE response amplitudes had the smallest average differences in the band 1.5 to 3 kHz (less than 2.5 dB). On the other hand, the largest difference, sometimes more than 9 dB, occurred at 6 kHz. This is consistent with reports by other authors [27,29,30,41,42]. The differences have been attributed to the acoustics of the external ear canal and interference between sound source and microphone [18,27].

Significantly greater differences were observed in terms of noise floors. The average values of this parameter were best (lowest) for the HearID DP and SmartDPOAE systems, and much higher (less good) for the Echoport ILO 292 USB I and Eclipse DPOAE20. Differences between the two reached more than 10 dB, especially in the band 1.5 to 4 kHz. Other authors [27,30] suggest that the differences may be due to different measurement and data analysis algorithms, the type of probe, and the type of ear tip. The position of the probe within the ear canal can have a significant effect, especially at higher frequencies [43].

In the busy ENT clinic environment (with possible ambient noise, frequent probe re-insertions, changing ear-canal conditions, time pressure), a system with a lower inherent noise floor offers a practical advantage. It is likely to lead to shorter test times, fewer repeat attempts, and higher confidence in the results for high-risk patients (e.g., those undergoing chemotherapy). Conversely, a system with a higher noise floor is likely to require more cautious interpretations or additional confirmatory tests.

The described differences in noise floors are directly related to the SNR parameter, values for which differed by up to 9 dB at some frequencies. However, although the SNR values differed between systems, it must be noted that the results for most individual DPOAE measurements were the same. That is, after applying the criterion of SNR > 6 dB for the presence or absence of an OAE signal as described in the methodology [44], all systems gave the same pass or fail result at particular frequencies, as noted by other authors [29,31]. Nonetheless, in an ototoxicity monitoring protocol, where there is a concern not just about pass/fail but also about small downward shifts in DPOAE amplitude or SNR over time, these inter-device differences matter. If a hospital switches device or combines data from different systems (for example across departments), an apparent “drop” in DPOAE level might in fact reflect system differences rather than cochlear change.

The shape of the DPOAE frequency response curves for all systems was similar to that reported in the literature [45]. The signal level increased from 1 kHz, reaching a first maximum at 1.5 kHz and a second at 3–4 kHz. For two of the systems, HearID DP and Echoport ILO 292 USB I, a third maximum was reached at 5–6 kHz. For all tested devices, the peak amplitude was reached at 3 kHz, and for Eclipse DPOAE20 an additional peak at 6 kHz. This phenomenon may be related to the occurrence of standing waves [30,46,47]. In addition, the differences in recorded DPOAE levels between systems may be due to the fact that the measurements were made at slightly different frequencies and rounded to a common frequency for comparison purposes [48].

The noise floors for the two systems—HearID DP and SmartDPOAE—were similar to those found in previous works [27,28,29,30].

Analysis of measurement stability before and after refitting the probe showed significant differences in standard error of measurement (SEM) values for each system and at each frequency tested. For the Echoport ILO 292 USB I system, SEMs ranged from 1.42 to 2.40 dB, for Eclipse DPOAE20 from 1.7 to 2.71 dB, for HearID DP from 0.99 to 2.88 dB, and for SmartDPOAE from 1.32 to 2.66 dB. In a review of several findings, Reavis and colleagues found SEMs ranging from 0.57 dB for low frequencies to 3.9 dB for high frequencies [17]. The authors attributed this to differences in group sizes, equipment calibrations, sealing tips, ear canal volumes, and standing waves [17], as well as depth of probe insertion [49].

In clinical terms, this suggests that when using any of these systems for serial monitoring (especially in settings where the probe must be removed and re-inserted, e.g., patient repositioning, changing ears, or cleaning the ear canal), clinicians should anticipate an inherent measurement noise of approximately 1 to 3 dB depending on frequency and system. When considering a drop in DPOAE amplitude of say 4 to 5 dB as clinically significant, one must know the system’s repeatability (SEM) and whether the same combination of device, protocol, and probe-fit was used.

A noteworthy finding here was that the mean differences in DPOAE amplitudes after refitting the probe were much lower than those reported by other authors. In most cases, the differences we found did not exceed 2 dB. For most systems tested, the worst repeatability was at 1 kHz (3.36 dB for the Echoport ILO 292 USB I) and at 8 kHz (3.76 dB for the IHS). Other authors have found much greater differences between DPOAE measurements, in some cases as much as 6 dB. We also confirmed that the best repeatability for measurements occurs in the band from 1.5 to 6 kHz [20,21,22,24,44,50,51,52]. Some reports recommend that the 6 kHz frequency should be excluded because of high fluctuations between measurements [22,44]; however, we found no differences for this frequency. This may be due to the smaller measurement sample or because of different measurement equipment.

## 5. Conclusions

Due to the large number of DPOAE measurement systems available on the market, it is important for those conducting tests, and when comparing them with results from other devices, to be aware that differences between measurements can be caused not only by pathological changes in the auditory system but also by differences between devices and the parameters used for setting up DPOAE recordings. When monitoring the effects of different factors on cochlear conditions, such as noise or ototoxic drugs, we therefore recommend always using the same device. More broadly, there is also a need for universal standards for OAE measurements that minimize cross-device differences.

Our results clearly demonstrate that DPOAE devices cannot be considered clinically interchangeable. Healthcare providers, researchers, and those framing standards should recognize that the device employed is a critical factor in ensuring consistency in DPOAE testing. Until industry-wide standardization is achieved, clinical decision-making needs to take into account device-specific factors. Most importantly, longitudinal monitoring programs need to prioritize measurement consistency over equipment convenience.

## Figures and Tables

**Table 1:** Measurement systems, the versions used in this study, and the DPOAE stimuli parameters. The actual 2F1 − F2 frequencies generated by the devices were rounded to 1, 1.5, 2, 3, 4, 6, and 8 kHz in order to make comparisons.

Manufacturer	System Name	Version	Probe Microphone	2F1 − F2 Frequency [kHz]	F2/F1 Ratio	F1 and F2 Levels
Otodynamics, Hatfield, UK	Echoport ILOv6 USB I	6.41.27.0	ILO probe	1, 1.5, 2, 3, 4, 6, 8	1.22	F1 = 65 dB SPL F2 = 55 dB SPL
Interacoustics, Middelfart, Denmark	Eclipse DPOAE20	1.03.1	Eclipse probe			
Mimosa Acoustics, Champaign, IL, USA	HearID DP	5.1.3.0	Etymotic Research ER-10C probe			
Intelligent Hearing Systems, Glenvar Heights, FL, USA	SmartDPOAE	4.70	Etymotic Research ER-10D probe			

**Table 2:** ICC, SEM, and MDC for two successive measurements of DPOAE amplitude, made before and after repositioning the probe. Key: ICC, intraclass correlation coefficient; SEM, standard error of measurement; MDC, minimum detectable change for 95% interval; *N* pass, number of ears passing the 6 dB criterion in each subgroup.

		1 kHz	1.5 kHz	2 kHz	3 kHz	4 kHz	6 kHz	8 kHz
Echoport ILOv6 USB I	ICC	0.87	0.88	0.90	0.92	0.91	0.88	0.90
	SEM	2.08	2.22	1.82	1.56	1.42	2.40	2.27
	MDC	5.77	6.16	5.05	4.32	3.94	6.66	6.30
	*N* pass	55	77	79	77	78	78	61
Eclipse DPOAE20	ICC	0.86	0.91	0.91	0.84	0.93	0.92	0.92
	SEM	2.71	1.98	1.75	2.38	1.70	2.19	2.24
	MDC	7.51	5.50	4.84	6.59	4.71	6.07	6.20
	*N* pass	77	84	82	84	83	79	60
HearID DP	ICC	0.94	0.95	0.91	0.95	0.97	0.94	0.86
	SEM	1.39	1.20	1.68	1.17	0.99	1.68	2.88
	MDC	3.84	3.32	4.66	3.24	2.74	4.64	7.98
	*N* pass	75	84	84	84	84	81	56
SmartDPOAE	ICC	0.82	0.78	0.90	0.91	0.93	0.96	0.83
	SEM	2.66	2.25	1.83	1.32	1.34	1.56	2.60
	MDC	7.38	6.24	5.08	3.66	3.73	4.32	7.20
	*N* pass	73	80	82	83	82	77	46

## Data Availability

The authors confirm that the data supporting the findings of this study are available within the article and its supporting materials.

## Supplementary Materials

The following supporting information can be downloaded at: https://www.mdpi.com/article/10.3390/jcm14228184/s1. Supplementary data: Echoport ILOv6 USB; Eclipse DPOAE20; HearID+DP; SmartDPOAE.

## Abbreviations

The following abbreviations are used in this manuscript:

DPOAE	distortion-product otoacoustic emissions
OAE	otoacoustic emission
SNR	signal-to-noise ratio
ICC	intraclass correlation coefficient
SEM	standard error of measurement
STD	standard deviation
MDC	minimum detectable change for 95% interval

## References

[1] Kemp D.T. (1978). Stimulated acoustic emissions from within the human auditory system. J. Acoust. Soc. Am..

[2] Konopka W., Pietkiewicz P., Zalewski P. (2000). Otoacoustic emission examinations in soldiers before and after shooting. Otolaryngol. Pol..

[3] Hendler B., Fiszer M., Śliwińska-Kowalska M. (2002). Zastosowanie emisji otoakustycznej wywołanej trzaskiem w monitorowaniu uszkodzeń słuchu spowodowanych hałasem. Otolaryngol. Pol..

[4] Lapsley Miller J.A., Marshall L., Heller L.M., Hughes L.M. (2006). Low-level otoacoustic emissions may predict susceptibility to noise-induced hearing loss. J. Acoust. Soc. Am..

[5] Helleman H.W., Dreschler W.A. (2012). Overall versus individual changes for otoacoustic emissions and audiometry in a noise-exposed cohort. Int. J. Audiol..

[6] Lonsbury-Martin B.L., Whitehead M.L., Martin G.K. (1991). Clinical applications of otoacoustic emissions. J. Speech Hear. Res..

[7] Gorga M.P., Neely S.T., Ohlrich B., Hoover B., Render J., Peters J. (1997). From laboratory to clinic: A large scale study of distortion product otoacoustic emissions in ear with normal hearing and ears with hearing loss. Ear Hear..

[8] Harris F.P., Lonsbury-Martin B.L., Stragner B.B., Coats A.C., Martin G.K. (1989). Acoustic distortion products in humans: Systematic changes in amplitude as a function of f2/f1 ratio. J. Acoust. Soc. Am..

[9] Gaskill S.A., Brown A.M. (1990). The behaviour of the acoustic disortion product 2f1-f2 from the human ear and its relation to auditory sensitivity. J. Acoust. Soc. Am..

[10] Lonsbury-Martin B.L., Martin G.K. (1990). The clinical utility of distortion-product otoacoustic emissions. Ear Hear..

[11] Pasdelou M.P., Byelyayeva L., Malmström S., Pucheu S., Peytavy M., Laullier H., Hodges D.D., Tzafriri A.R., Naert G. (2024). Ototoxicity: A high risk to auditory function that needs to be monitored in drug development. Front. Mol. Neuroci..

[12] Konrad-Martin D., Reavis K.M., McMillan G.P., Dille M.F. (2012). Multivariate DPOAE metrics for identifying changes in hearing: Perspectives from ototoxicity monitoring. Int. J. Audiol..

[13] Reavis K.M., Phillips D.S., Fausti S.A., Gordon J.S., Helt W.J., Wilmington D., Bratt G.W., Konrad-Martin D. (2008). Factors affecting sensitivity of distortion-product otoacoustic emissions to ototoxic hearing loss. Ear Hear..

[14] Knight K.R., Kraemer D.F., Neuwelt E.A. (2005). Ototoxicity in children receiving platinum chemotherapy: Underestimating a commonly occurring toxicity that may influence academic and social development. J. Clin. Oncol..

[15] Kemp D.T. (2002). Otoacoustic emissions their origin in cochlear function, and use. Br. Med. Bull..

[16] Ozimek E. (2005). Emisje otoakustyczne w aspekcie fizycznym i klinicznym. Postępy Chir. Głowy Szyi..

[17] Reavis K.M., McMillan G.P., Dille M.F., Konrad-Martin D. (2015). Meta-Analysis of Distortion Product Otoacoustic Emission Retest Variability for Serial Monitoring of Cochlear Function in Adults. Ear Hear..

[18] Siegel J.H. (1994). Ear-canal standing waves and high-frequency sound calibration using otoacoustic emission probes. J. Acoust. Soc. Am..

[19] Charaziak K.K., Shera C.A. (2017). Compensating for ear-canal acoustics when measuring otoacoustic emissions. J. Acoust. Soc. Am..

[20] Dreisbach L.E., Long K.M., Lees S.E. (2006). Repeatability of high-frequency distortion-product otoacoustic emissions in normal hearing adults. Ear Hear..

[21] Franklin D.J., McCoy M.J., Martin G.K., Lonsbury-Martin B.L. (1992). Test/Retest Reliability of Distortion-Product and Transiently Evoked Otoacoustic Emissions. Ear Hear..

[22] Roede J., Harris F.P., Probst R., Xu L. (1993). Repeatability of distortion product otoacoustic emissions in normally hearing humans. Audiology.

[23] Beattie R.C., Kenworthy O.T., Luna C.A. (2003). Immediate and shortterm reliability of distortion-product otoacoustic emissions. Int. J. Audiol..

[24] Ng I.Y., McPerson B. (2005). Test-retest reliability of distortion product otoacoustic emissions in the 1 to 7 kHz range. Audiol. Med..

[25] Dreisbach L.E., Zettner E., Chang Liu M., Meuel Fernhoff C., MacPhee I., Boothroyd A. (2018). High-Frequency Distortion-Product Otoacoustic Emission Repeatability in a Patient Population. Ear Hear..

[26] Pilka E., Jedrzejczak W.W., Kochanek K., Skarzynski H. (2019). Variability of high-frequency distortion product otoacoustic emissions measured by the SmartOAE device: Preliminary study. J. Hear. Sci..

[27] Hornsby B., Kelly T., Hall J.W. (1996). Normative data five FDA-approved distortion product OAE system. Hear. J..

[28] Parthasarathy T.K., Klostermann B. (2001). Similarities and differences in distortion-product otoacoustic emissions among four FDA-approved devices. J. Am. Acad. Audiol..

[29] Pilka E., Jedrzejczak W.W., Olszewski L., Skarzynski H. (2016). High-frequency distortion product otoacoustic emissions measured by two systems: An example of a subject with normal hearing. J. Hear. Sci..

[30] Cañete O.M., El-Haj-Ali M., Fereczkowski M. (2024). Comparison of Two Clinical Devices for the Measurement of Distortion Product Otoacoustic Emissions in Normal-Hearing Adults. J. Audiol. Otol..

[31] Jedrzejczak W.W., Gos E., Pilka E., Skarzynski P.H., Skarzynski H., Hatzopoulos S. (2021). Pitfalls in the detection of Hearing Loss via Otoacoustic Emissions. Appl. Sci..

[32] Jerger J. (1972). Clinical experience with impedance audiometry. Arch. Otoloaryngol..

[33] Liden G., Harford E., Hallen O. (1974). Automatic tympanometry in clinical practice. Audiology.

[34] Olusanya B.O., Davis A.C., Hoffman H.J. (2019). Hearing loss grades and the International classification of functioning, disability and health. Bull. World Health Organ..

[35] Harrell R.W., Katz J. (2002). Pure tone evaluation. Handbook of Clinical Audiology.

[36] Jedrzejczak W.W., Pilka E., Pastucha M., Kochanek K., Skarzynski H. (2023). Extended High Frequency Thresholds and Their Relationship to Distortion Product Otoacoustic Emissions, Hearing Acuity, Age, Gender, Presence of Spontaneous Otoacoustic Emissions, and Side of Measurement. Appl. Sci..

[37] Hall J.W., Swanepoel W. (2010). Objective Assessment of Hearing.

[38] Christensen L.A. (2000). A Universal Pass/Refer Criterion for DPOAE: Is it Possible?. Hear. Rev..

[39] Hall J.W. (2000). III. Hand Book of Otoacoustic Emissions.

[40] Harris F.P., Probst R., Robinette M.S., Glattke T.J. (2002). Otoacoustic Emissions and Audiometric Outcomes. Otoacoustic Emissions Clinica Applications.

[41] Pilka E., Jedrzejczak W.W., Trzaskowski B., Skarzynski H. (2014). Variability of Distortion Product Otoacoustic Emissions at 10, 12 and 16 kHz: A preliminary study. J. Hear. Sci..

[42] Gorga M.P., Neely S.T., Bergman B.M., Beauchaine K.L., Kaminski J.R., Peters J., Schulte L., Jesteadt W. (1993). A comparison of transient-evoked and distortion product otoacoustic emissions in normal-hearing and hearing-impaired subjects. J. Acoust. Soc. Am..

[43] Zebian M., Hensel J., Fedtke T., Vollbort S. (2011). Interpretation of distortion product otoacoustic emissions at higher frequencies. J. Hear. Sci..

[44] Wagner W., Heppelmann G., Vonthein R., Zenner H.P. (2008). Test-retest repeatability of distortion product otoacoustic emissions. Ear Hear..

[45] Dunckley K.T., Dreisbach L.E. (2004). Gender effects on high frequency distortion product otoacoustic emissions in humans. Ear Hear..

[46] Prive P., Fitzgerald T., Katz. J., Chasin M., English K., Hood L.J., Tillery K. (2025). Otoacoustic emissions. Handbook of Clinical Audiology.

[47] Carter L., Williams W., Seeto M. (2015). TE and DP otoacoustic emissions data from an Australian cross-sectional hearing study. Int. J. Audiol..

[48] Shaffer L.A., Withnell R.H., Dhar S., Lilly D.J., Goodman S.S., Harmon K.M. (2003). Sources and mechanisms of DPOAE generation: Implications for the prediction of auditory sensitivity. Ear Hear..

[49] Souza N.N., Dhar S., Neely S.T., Siegel J.H. (2014). Comparison of nine methods to estimate ear-canal stimulus levels. J. Acoust. Soc. Am..

[50] Zhao F., Stephens D. (1999). Test-retest variability of distortion-product otoacoustic emissions in human ears with normal hearing. Scand. Audiol..

[51] Keppler H., Dhooge I., Maes L., D’haenens W., Bockstael A., Philips B., Swinnen F., Vinck B. (2010). Transient-evoked and distortion product otoacoustic emissions: A short-term test-retest reliability study. Int. J. Audiol..

[52] Sockalingam R., Lee Choi J., Choi D., Kei J. (2007). Test-retest reliability of distortion-product otoacoustic emissions in children with normal hearing: A preliminary study. Int. J. Audiol..

